# Emergent Transcriptomic Technologies and Their Role in the Discovery of Biomarkers of Liver Transplant Tolerance

**DOI:** 10.3389/fimmu.2015.00304

**Published:** 2015-06-22

**Authors:** Sotiris Mastoridis, Marc Martínez-Llordella, Alberto Sanchez-Fueyo

**Affiliations:** ^1^Institute of Liver Studies, King’s College Hospital, London, UK

**Keywords:** liver, transplant, tolerance, biomarkers, transcriptome, gene, transplantomics

## Abstract

Liver transplantation offers a unique window into transplant immunology due, in part, to the considerable proportion of recipients who develop immunological tolerance to their allograft. Biomarkers are able to identify and predict such a state of tolerance, and thereby able to establish suitable candidates for the minimization of hazardous immunosuppressive therapies, are not only of great potential clinical benefit but might also shed light on the immunological mechanisms underlying tolerance and rejection. Here, we review the emergent transcriptomic technologies serving as drivers of biomarker discovery, we appraise efforts to identify a molecular signature of liver allograft tolerance, and we consider the implications of this work on the mechanistic understanding of immunological tolerance.

## Introduction

The liver represents a unique window to the immune system. Unlike other transplanted organs, it exhibits immunoregulatory, tolerogenic properties, enabling an allograft to be more readily spontaneously accepted. The phenomenon of operational tolerance, i.e., stable allograft function in spite of complete discontinuation of immunosuppressive therapy, while rarely achieved in cases of renal transplantation, for instance, is relatively commonplace in liver transplant recipients. Indeed, the prevalence of operational tolerance following liver transplantation appears to be far greater than previously appreciated. Until recent clinical trial evidence to the contrary, an average estimate was that approximately 20% of liver allograft recipients were able to successfully be weaned off immunosuppression, and thereby to achieve a state of induced operational tolerance (1, 2). Benítez and colleagues, however, showed that a remarkable 42% of 98 liver allograft recipients undergoing weaning of immunosuppression achieved operational tolerance. Furthermore, the propensity to tolerance was noted to develop over time, with those who had had their graft for 10.6 years or more achieving tolerance in 79.2% of cases (3). While these results should be taken with some caution, the inescapable implication is that a significant proportion of liver transplant recipients, particularly if in the second decade of graft survival, are unnecessarily subjected to immunosuppressive therapy and the significant risks associated with it. The incentive, therefore, to search for a biomarker by which to identify patients amenable to drug minimization, becomes clear. Furthermore, the pursuit of such biomarkers might aid in the fuller characterization of the immunological phenotype associated with tolerance, and so offer a mechanistic understanding of the processes by which tolerance is achieved and might be induced.

Major advances have been made in recent years in the fields of genetic and molecular biology. Large international collaborations such as the human genome and proteome projects enabled further technological developments of high-throughput technologies. Broadly, with new technologies, arise new investigative paradigms. Reductionist scientific approaches have been overtaken, to an extent, by the generation of vast biological datasets enabling the study of complete sets of molecules. The umbrella neologism “omics” has appeared in order to describe these changes and to classify emerging fields – metabolomics, proteomics, transcriptomics, and so on. The systems biology approach has developed to offer a computational and mathematical framework enabling the integration and analysis of data from these seemingly disparate fields. The application of these developing fields to the arena of transplantation, with a view to the personalized treatment of patients, has recently been dubbed as “transplantomics” (4, 5).

Regarding the identification of biomarkers of tolerance, of all the emerging work in transplantomics, the high-throughput measurement of the transcriptome has shown the greatest promise and formed a focus of research. Here, we review the application of transcriptomic technologies to the unique window proffered by liver transplantation tolerance. We set out an overview of the technologies and of the associated analytical tools. We review recent progress in developing novel biomarkers of tolerance, and look at their application in trials of immunosuppression withdrawal. We discuss the limitations and pitfalls associated with high-throughput transcriptomic research. Finally, we consider the implications of these tools on our mechanistic understanding of operational tolerance and how this might guide future therapeutic developments.

## Principles of Transcriptome Analysis

The “transcriptome” describes the complete set of messenger RNA (mRNA) and non-coding RNA (ncRNA) transcripts, which include micro RNA (miRNA), small nuclear RNA (snRNA), and small nucleolar RNA (snoRNA) among others. Comprehensive understanding of the transcriptome must also take into consideration further complexities – splicing isoforms, gene-fusion transcripts, post-translational modifications, and epigenetic controls for example. Thus, “Transcriptomics,” can be understood as the large-scale study of transcriptional products as well as their regulation and modification (5, 6).

### Transcriptome profiling

Transcriptome profiling can be subdivided into two general approaches for simplicity – the candidate gene strategy focuses on single gene transcripts, while high-throughput approaches allow for the simultaneous measurements of thousands of transcripts. The first candidate gene-based studies utilized the Northern Blot (7). This method fixed RNA on a solid support, following its separation by electrophoresis, and then the presence and abundance of the fixed RNA species of interest were deduced by hybridization with complementarily labeled radioactive nucleic acid probes. The low throughput and requirement of large quantities of input RNA made this technique cumbersome. Reverse transcriptase polymerase chain reaction (RT-PCR) is now the method most commonly used for candidate gene transcript measurement and has broad applications in the clinical setting (8). In this approach, mRNA is reverse transcribed to complementary DNA (cDNA) and amplified with primers specific for the gene of interest using PCR. Quantitative measures of mRNA abundance are made possible by monitoring the accumulation of PCR product (9). While the method requires only small quantities of input RNA, is robust, cost-effective, and rapid, the throughput remains in the order of hundreds of known transcripts at a time and so is not amenable to transcriptome-wide investigations (10).

Microarray technology, on the other hand, has enabled the rapid, simultaneous measurement of the whole transcriptome. mRNA is hybridized to an array of oligonucleotide or cDNA probes that are robotically spotted onto a solid support chip, thereby allowing the identity of each probe to be defined by its location. Hybridization intensity to a particular probe is related to the abundance of corresponding transcript (11, 12). Microarray technology has been applied to the gamut of transplantation biology over the last decade, including studies of acute and chronic rejection, and more relevant herein, the understanding of immune tolerance and identification of biomarkers. Microarrays have become the best standardized, most affordable, and widely accessible of the high-throughput omics technologies (13).

### Microarray experimental design and analysis

A typical microarray generates expression levels for thousands of genes, thereby producing vast quantities of data. The major challenge is to analyze and understand these data, to distinguish true from misleading signals on the one hand, and to uncover clinically relevant findings on the other. The steps typically involved in a microarray experiment are (i) experimental design, (ii) sample preparation and processing, and (iii) data analysis and interpretation. Careful experimental design is crucial. It depends heavily on the array technology used and, of course, on the research objectives (14, 15). Objectives are often characterized as either “class comparison” or “class prediction” (16). In this setting, the former describes attempts to identify genes differentially expressed between operationally tolerant recipients and another comparison group. The latter involves the development of multi-gene formulae able to predict which patients might exhibit tolerance based on their expression profiles. The high-dimensional datasets generated cannot be adequately analyzed with conventional comparative statistics. The complexity of analysis and the potential pitfalls require a team approach and a good understanding of the relevant software required for the steps of quality control, normalization, clustering, classification, and pathway analyses (8, 17).

### More data herald more challenges…

Rigorous quality control criteria help to ensure high quality data collection from arrays that are reproducible and comparable. The MicroArray Quality Control project (MAQC), an unprecedented, community-wide effort to appraise microarray reliability and quality control metrics, reported that, with careful experimental design and appropriate data transformation and analysis, data can be reproducible and comparable across laboratories, institutions, and researchers (18). A number of commercial software packages have been developed to aid the quality control process (19–22). Specialist software is also available to aid with data normalization, a crucial step in the conversion of raw data into scaled relative expression levels (8, 23, 24). Statistical packages are also utilized for calculating differential expression, controlling for false positives, selecting significance cut-offs, the clustering of genes thought to be similar or co-regulated, and final pathway analyses, enabling the identification of gene sets associated with specific biological functions (25–27).

Much of the complexity involved in the statistical analyses stems not only from the high numbers of genes measured per sample (“curse of dimensionality”) but also the disproportion between this and the limited numbers of samples available for testing (“curse of scarcity”) – a difficulty often faced in biomarker research. This is overcome, in part, through adjusted *p*-values (q-value) such as false discovery rates (5). Tools for analysis are often free to download and now widely used. They include significance analysis of microarrays (SAM), GenePattern, and GenMAPP (5). Despite robust analytical tools, an undiscerning researcher can erroneously use data to “discover” sets of genes that are able to differentiate the samples on which the gene algorithm modeling was based even when the data are completely random. This problem should be circumvented by ensuring that a gene model is tested on a validation group that is independent from the training set used to create the model in the first place. This approach is more desirable than cross-validation techniques sometimes employed (28–30). Further, technical validation of microarray results on a different transcriptional platform, usually RT-PCR, is also recommended to minimize inter- or intra-platform variability in hybridization noise that may arise between batches or laboratories.

In order to verify the reproducibility of analyses and to corroborate clinical validity, public microarray databases serve as essential repositories. In the transplant setting, where studies often include only small numbers of recipients, these resources are especially important. The Functional Genomics Data (FGED) Society (formerly the MGED Society), a non-profit, volunteer run organization promoting the sharing of high-throughput research data, helped to define the Minimum Information About a Microarray Experiment (MIAME) guidelines for data content standards. The Society also set the standard data exchange format, known as the Microarray Genetic Expression Markup Language (MAGE-ML). Thorough reviews of the numerous databases in existence have been set out in the literature (31).

Microarray data output is necessarily dependent on the quality of the original biological samples. RNA is considerably more susceptible to rapid enzymatic degradation than DNA, thereby making efficient processing and appropriate storage using robust protocols essential. Microarrays offer snapshots of gene expression. The kinetics of transcripts and the variability of changing levels of expression in relation to their baseline remain little understood and so are not amenable to statistical interpretation (32, 33). Matters are further complicated by tissue heterogeneity, as is the case in blood samples for instance. This heterogeneity makes anatomical detail in the microarray approach difficult, in that it is difficult to know which cells’ gene expression profiles are being analyzed. Cell sorting and microdissection are ways to tackle this difficulty, as is the application of statistical deconvolution methods such as the cell-specific significance analysis of microarrays (csSAM) (34). While peripheral blood has been at the forefront of efforts to identify biomarkers, the possibility of interrogating RNA extracted from paraffin embedded biopsies is a useful addition to investigative efforts.

It becomes clear then, that to discern biological fact from mere noise, it is essential that due attention is paid to the analytical complexities involved in microarray interpretation. Although, as we will see, microarray profiling has yielded important data in the pursuit of biomarkers of tolerance, and the technology is becoming more commonplace in transplantation research, the promise of emerging next-generation sequencing (NGS) technologies is likely to eclipse many microarray applications. In essence, NGS involves the sequential identification of the bases of small fragments of DNA from signals, which are emitted when each fragment is re-synthesized from a DNA template strand. By extending this process across millions of reactions in parallel, the technology enables rapid sequencing of large stretches of DNA base-pairs spanning entire genomes (35).

In part, the promise of NGS stems from sidestepping some of the aforementioned problems inherent in microarray technology. NGS is highly reliable, and has greater dynamic range as it directly quantifies discrete digital sequencing readouts as opposed to relying on hybridization steps. Loss of specificity due to cross-hybridization is controlled; the detection of rare and low abundance transcripts is made more achievable; the unbiased detection of novel transcripts is made possible since the need for transcript-specific probes utilized by microarray become redundant; and errors in probe design, which are relatively common in microarray chips, are avoided. In addition to these technical considerations, NGS technology is advancing at such a pace that the prospect of “sequencing everything” (genome, epigenome, transcriptome) in a timely and cost-effective manner is well within reach. In the 4 years between 2007 and 2011, a single sequencing run’s output increased 1000×, far outstripping Moore’s law, while the cost of sequencing the entire genome has fallen from over 150,000 USD in 2009, to less than 5000 USD in 2014 (36). Of course, NGS presents its own technological and bioinformatics challenges – which have been comprehensively reviewed elsewhere (37, 38).

## Identification of Tolerance Biomarkers

Much hope has been placed upon transcriptomic technologies as the drivers of a “new era of individualized therapy” (4). The application of these technologies in the discovery of novel diagnostic and predictive markers has spanned diverse transplantation research fields, including the development of predictors of allograft risk, the identification of biomarkers of acute and chronic allograft injury, the assessment of organ suitability and viability during the preservation period, and forming the focus here, the discovery of biomarkers of tolerance.

Much of this work is in its infancy; transcriptomic investigation of biomarkers of liver allograft tolerance began less than a decade ago (39). Already though, biomarker-based diagnostic tests have gained regulatory approval and have reached the market (40–42). A diagnostic kit based on an 11-transcript set identified with microarray technology is used to non-invasively identify rejection in heart transplant recipients (41). As we will see, biomarkers of liver transplant tolerance have also yielded extremely promising results showing good potential for clinical translation in the near future.

Martínez-Llordella and colleagues were the first to use microarray technology for the gene expression profiling of blood samples from operationally tolerant liver transplant patients (39). This retrospective, cross-sectional study compared 16 operationally tolerant recipients to 16 recipients failing to undergo immunosuppression withdrawal, and found 462 positively and 166 negatively regulated genes. Functional analysis revealed that tolerance expression profiles were enriched in gamma-delta T (γδ T) cells and natural killer (NK) cells (see Table 1). Genes involved in mRNA processing, protein biosynthesis, DNA repair, cell cycle control, Interleukin 2 receptor signaling, and transcription regulation were also noted to be differentially expressed. While this was a first step toward proof of principle, there were considerable methodological limitations. One difficulty common to all studies of tolerant patients is the selection of an appropriate control group. Stable transplant patients are sometimes used as a control, but the immunosuppressive medications they receive may skew any comparative interpretations. Another approach is to use healthy controls to circumvent the concerns with immunosuppressive therapy, but in this case the absence of transplantation becomes a significant limitation in itself. Without a perfect control population, one reasonable approach is to use multiple control groups. In an attempt to address other methodological limitations with their first study, Martínez-Llordella’s group followed up with a more robust analysis of a larger cohort of patients and incorporated both training and validation sets, as well as the necessary cross-validation checkpoint procedures (43). Of 1932 differentially expressed genes identified in this follow-up study, RT-PCR validation of 68 promising candidate genes was performed with good correlation shown between platforms. Utilizing a novel modeling approach based on the misclassified penalized posterior (MiPP) algorithm, three optimally parsimonious gene signatures were identified, containing 2, 6, and 7 genes, respectively, and altogether comprising 12 different genes (see Table 1). These signatures were shown to be capable of accurately predicting the clinical status not only of the group of recipients from whom they were derived but also of an independent validation cohort of 23 patients. When these gene signatures were evaluated against a cohort of stable recipients on maintenance immunosuppression, they predicted that 26% of these patients would be tolerant; a prediction that is roughly equivalent to the prevalence of tolerance indicated by the literature (1, 44, 45).

**Table 1:** **Studies using microarray transcriptomic profiling to identify biomarkers of liver transplant tolerance**.

Study	Study population	Tissues analyzed	Microarray platform	Summary/significance	Reference
Martínez-Llordella (2007)	16 OLTT; 16 nOLTT	Blood	Affimetrix	Retrospective, cross-sectional study	(39)
				462 up-regulated, 166 down-regulated genes identified	
				OLTT expression profiles enriched in gamma-delta T cells and natural killer cells (CD94, NKG2D, NKG7, TRD@, KLRC1, KLRC2, KLRB1, CD160)	
Kawasaki (2007)	11 OLTT; 11 HV	Blood	Agilent	Retrospective, cross-sectional study	(46)
				627 up-regulated and 90 down-regulated genes identified	
				No independent data validation steps performed	
Martínez-Llordella (2008)	28 OLTT; 33 nOLTT	Blood	Affimetrix	Retrospective, cross-sectional study	(43)
				Identification of three gene signatures, containing 2, 6, and 7 genes, respectively, and altogether comprising 12 different genes (KLRF1, SLAMF7, NKG7, IL2RB, KLRB1, FANCG, GNPTAB, CLIC3, PSMD14, ALG8, CX3CR1, RGS3)	
Lozano (2011)	12 OLTT; 12 nOLTT; 12 OKTT; 12 nOKTT; 12 HV	Blood	Affimetrix	Retrospective, cross-sectional study, multicenter study	(47)
				Enrichment of B cell-related transcript in OKTT, stable over time and across cohorts. Enrichment in natural killer cells in OLTT. Liver and kidney tolerant recipients exhibited distinct transcriptional and cell phenotypic patterns with little overlap	
Bohne (2012)	33 OLTT; 42 AR	Blood and biopsy	Affimatrix	Prospective, multicenter trial of immunosuppressive withdrawal in liver transplant recipients	(48)
				Enrichment of natural killer and gamma-delta cell transcripts corroborated	
				Accurate prognostic model developed using intragraft expression profiles, mainly enriched with genes involved in iron homeostasis	
Li (2012)	Pediatric: 16 OLTT; 19 nOLTT; 6HV; 22 STA; 20 MIS	Blood	Affimetrix and Agilent	Amalgamation of publically available gene-expression data, and data generated in two US centers of pediatric liver transplant recipients	(49)
	Adult: 17 OLTT; 21 nOLTT; 19 STA			Identification of 13-gene signature, of high predictive accuracy, and independent of recipient age, donor type, and concomitant viral infection	
				Enriched in natural killer cell transcripts (SENP6, FEM1C, ERBB2, AKR1C3, MAN1A1, UBAC2, GPR68, NFKB1, MAFG, BT3G, ASPH, PTBP2, PDE4DIP)	

*OLTT, patients exhibiting operational liver transplant tolerance; nOLTT, patients not achieving a state of operational liver transplant tolerance; OKTT, patients exhibiting operational kidney transplant tolerance; nOKTT, patients not achieving a state of operational kidney transplant tolerance; HV, healthy volunteer; STA, stable under standard immunosuppressive therapy; MIS, minimally immunosuppressed; AR, acute rejection*.

Mechanistic interpretations of these findings were hampered by the retrospective study design and the lack of simultaneous molecular analyses of allograft tissue. These issues were addressed in a prospective, multi-center immunosuppression withdrawal trial in liver transplant recipients, as reported by Bohne and colleagues (48). Of 75 recipients completing the trial, 42 underwent rejection, while 33 were successfully weaned off immunosuppression, thereby achieving a tolerant state. Microarray and RT-PCR analyses of both peripheral blood and the grafts themselves were conducted. While previous conclusions regarding peripheral blood mononuclear cell (PBMC) enrichment in NK and γδ cells were corroborated, of special interest is the fact that, in side-by-side comparisons, liver tissue-derived transcriptional signatures proved more robust, accurate, and reproducible than PBMC derived signatures. The intragraft expression profile was mainly enriched with genes involved in iron homeostasis, and showed no overlap with genes identified from PBMC. The role of iron redistribution is a well-established antimicrobial strategy and has been shown to play a significant part in pathogenic infection of the liver, where iron overload is associated with poorer outcomes (50–52). Whether this is a property mediated through effects on pathogen growth, or on the host immune response itself is unclear. What this study is first to highlight, though, is the possibility that the dampening of alloreactive immune responses required for the establishment of tolerance may be dependent on the iron-store status of the allograft.

Liver biopsy tissue was also analyzed in a more recent study by Zhao and colleagues looking at a cohort of pediatric patients (53). While previous work had already identified the enrichment of γδ T cell subpopulations and the genes associated with their expression in the peripheral blood of tolerant recipients, Zhao’s group examined these cells at the transcriptional level within the graft itself. Two prominent subsets of γδ T cells have been defined based on their δ chain – Vδ1 and Vδ2 T cells. Vδ2 cells are normally the predominant subset in blood and are involved in the inflammatory response. Vδ1 cells normally reside and are predominant in mucosal surfaces, possess potent immunoregulatory and suppressive capacities, and have been shown to emerge into the peripheral blood to a degree, which gives them predominance over Vδ2 cells in tolerant liver transplant recipients (39, 54). Zhao et al. showed that Vδ1 cells also accumulated within the grafts of operationally tolerant recipients in an antigen driven process, and that the complementarity-determining region 3 (CDR3) sequence of the δ chain of these Vδ1 cells specifically undergoes oligoclonal expansion, thereby suggesting that tolerance might be identified through sequencing analysis of these intragraft cells.

In the largest analysis of transcriptomic data pertaining to transplant tolerance, Li and colleagues extended previous work by developing a tolerance signature independent of recipient age and donor source, cause of end-stage liver disease, or concomitant viral infection (49). This was achieved through the amalgamation of living and deceased donor and pediatric, as well as adult data from across different clinical centers. The 13-gene tolerance signature identified (Table 1) was highly associated with NK cells, corroborating earlier work, and proved to have striking predictive accuracy, exhibiting 100% sensitivity and 83% specificity. This degree of predictive capacity would appear to obviate the need for the biopsy derived gene signatures, thought to be of superior utility as biomarkers of tolerance in earlier studies (43).

The benefits of identifying robust non-invasive biomarkers over those derived from biopsy tissue are self-evident. Non-coding transcripts such as miRNAs have been shown to be more stable in peripheral blood than mRNA, have been shown to be implicated in the control of genes relevant to alloreactive immune responses, and with the advent of NGS techniques offer the promise of novel PBMC-derived tolerance signatures (55, 56). Using miRNATaqman low-density arrays targeting 381 human miRNAs, Danger et al. reported on the modulation of expression of eight miRNAs in peripheral blood samples, nine tolerant kidney transplant recipients as compared to 10 patients with stable renal function under immunosuppression (57). They noted that B cells from the operationally tolerant group overexpressed miR-142-3p, and that this expression was not modulated by immunosuppression. The stability of miRNA in biofluids allowed Lorenzen et al. to investigate miRNA levels in the urine of a small retrospective cohort of kidney transplant recipients, and to identify miR-210 as a reliable marker of acute rejection and predictor of long-term graft function (58). In a multicentre cohort of renal allograft recipients, Suthanthiran and colleagues prospectively validated a three-gene urinary mRNA signature [interferon inducible protein 10 (IP-10) mRNA, 18S rRNA, and CD3ε mRNA] (59). Their results represent a major step toward achieving non-invasive diagnosis and prediction of acute allograft rejection, and highlight the utility of pursuing biomarkers across varied tissue and biofluid samples. The success of miRNA biomarkers in studies of renal allograft tolerance and rejection has helped to instigate some early work in rodent models of liver transplant tolerance, while human studies are still awaited (60, 61).

As highlighted by miRNA biomarkers, it would be remiss to conclude this review of transcriptomic research into biomarkers of *liver* transplant tolerance without reference to the important cross-fertilization of ideas, of methodological approaches, and of data and sample sharing with research groups investigating *kidney* transplantation tolerance. Transcriptomic research into kidney transplantation faces some unique challenges, the scarcity of patients able to achieve operational tolerance being one important example. Fewer than 200 cases of kidney operational tolerance have been described over the last 40 years (62). Nevertheless, with the successful development of research consortia in this field, a number of transcriptional studies have been successfully undertaken (47, 63–69). Very broadly, these reports presented gene lists converging toward a B cell signature of tolerance, and in so doing corroborated other data showing that both the percentage and the absolute number of B cells are increased in operationally tolerant kidney allograft recipients (64, 65, 70, 71). Despite efforts to coordinate these studies, reports on kidney transplant tolerance have been extremely heterogeneous in terms of the techniques used, the controls groups drawn upon, and the various clinical profiles of the patients studied. Unsurprisingly then, overlap between the gene-markers identified between research groups has been poor, raising questions about their reliability and about their eventual applicability in clinical contexts (72). Similarly, while identifying shared features of tolerance between kidney and liver transplant recipients would be helpful in finding common mechanistic processes underpinning tolerance induction, in developing therapeutic strategies, and in identifying novel biomarkers, it is the case that comparisons of data from disparate studies can be problematic. Array platforms often vary significantly in the probes they have in common; lymphochip and affymetrix chips, for instance, have only a few probes in common (43). Furthermore, in their direct comparison, employing the same transcriptional technology, Lozano et al. revealed an absence of significant overlap in blood phenotypic and transcriptional patterns between operationally tolerant liver and kidney recipients (47). Nevertheless, in recent work, the power of transplantomic technologies coupled with novel statistical techniques have helped to overcome many of these difficulties. This was exemplified by the recent identification of a common rejection molecule (CRM) across multiple transplanted organs (liver, kidney, heart, and lung) by Khatri and colleagues, who were able to compare and integrate data from several transcriptional studies by meta-analysis (73). The CRM consists of an 11-gene signature able to diagnose acute rejection with high sensitivity and specificity and could accurately predict future injury to a graft across all four organs. In recent months, a similar methodological approach was applied to integrate five of the disparate kidney transcriptional datasets aforementioned, in order to define a robust gene signature of operational tolerance (72). The meta-analytical methodology was able to reconcile the lack of overlap between the five studies, and to identify a gene-signature involving proliferation of B and CD4 T cells, and inhibition of CD14 monocytes. This gene signature, narrowed down to 20 biomarkers, underwent full cross validation, and was shown to be highly predictive in new samples and new patients, independent of the array technology used. It is critical that similar meta-analyses are performed on the liver tolerance datasets discussed here. The proof of the clinical utility of all these predictive biomarker sets rests on their successful application in prospective studies of biomarker-targeted immunosuppression weaning within a randomized, controlled setting. This precisely, is the purpose of a large, European trial currently underway called “BIOmarker-Driven personalized IMunosuppression,” or BIO-DrIM (www.biodrim.eu).

## Conclusion

The unique characteristics of the liver transplant setting, alongside the technological advances in transplantomic disciplines, which have enabled the discrimination of operational tolerance at a molecular level, present researchers with the opportunities to decipher the immunological mechanisms underlying drug-free allograft survival and to develop therapeutic targets aimed toward tolerance induction strategies.

The understanding that a large proportion of liver transplant recipients, particularly those living with their graft for a number of years, are over-immunosuppressed, must act to incentivize the translation of biomarker discovery into everyday clinical practice.

Emergent technologies, including next generation sequencing, must be capitalized upon to provide insights into normal, pathological, and pharmacological processes. As the diverse omics fields become more elaborate and produce ever more data, the collaboration between researchers, laboratories, hospitals and other institutions, and the integration of clinical and molecular data become essential to the pursuit of advancing the field of transplantation and developing personalized therapy.

## Author Contributions

SM, MM-L, and AS-F all contributed to the conception, drafting, critical revision, and final approval of this work. All are accountable for its content.

## Conflict of Interest Statement

The authors declare that the research was conducted in the absence of any commercial or financial relationships that could be construed as a potential conflict of interest.

## References

[1] Heidt S Wood KJ Biomarkers of operational tolerance in solid organ transplantation. Expert Opin Med Diagn (2012) 6:281–93. 10.1517/17530059.2012.680019 22988481 PMC3442251

[2] Sawitzki B Pascher A Babel N Reinke P Volk H-D. Can we use biomarkers and functional assays to implement personalized therapies in transplantation? Transplantation (2009) 87:1595–601. 10.1097/TP.0b013e3181a6b2cf 19502949

[3] Benítez C Londoño M-C Miquel R Manzia T-M Abraldes JG Lozano J-J Prospective multicenter clinical trial of immunosuppressive drug withdrawal in stable adult liver transplant recipients. Hepatology (2013) 58:1824–35. 10.1002/hep.26426 23532679

[4] Sarwal MM Benjamin J Butte AJ Davis MM Wood K Chapman J. Transplantomics and biomarkers in organ transplantation: a report from the first international conference. Transplantation (2011) 91:379–82. 10.1097/TP.0b013e3182105fb8 21278631 PMC3762499

[5] Naesens M Sarwal MM. Molecular diagnostics in transplantation. Nat Rev Nephrol (2010) 6:614–28. 10.1038/nrneph.2010.113 20736923

[6] Martin JA Wang Z. Next-generation transcriptome assembly. Nat Rev Genet (2011) 12:671–82. 10.1038/nrg3068 21897427

[7] Alwine JC Kemp DJ Stark GR. Method for detection of specific RNAs in agarose gels by transfer to diazobenzyloxymethyl-paper and hybridization with DNA probes. Proc Natl Acad Sci U S A (1977) 74:5350–4. 10.1073/pnas.74.12.5350 414220 PMC431715

[8] Mueller TF Mas VR. Microarray applications in nephrology with special focus on transplantation. J Nephrol (2012) 25:589–602. 10.5301/jn.5000205 22972670

[9] Becker-André M Hahlbrock K. Absolute mRNA quantification using the polymerase chain reaction (PCR). A novel approach by a PCR aided transcript titration assay (PATTY). Nucleic Acids Res (1989) 17:9437–46. 10.1093/nar/17.22.9437 2479917 PMC335144

[10] VanGuilder HD Vrana KE Freeman WM. Twenty-five years of quantitative PCR for gene expression analysis. Biotechniques (2008) 44:619–26. 10.2144/000112776 18474036

[11] Pozhitkov AE Tautz D Noble PA. Oligonucleotide microarrays: widely applied poorly understood. Brief Funct Genomic Proteomic (2007) 6:141–8. 10.1093/bfgp/elm014 17644526

[12] Schena M Shalon D Davis RW Brown PO. Quantitative monitoring of gene expression patterns with a complementary DNA microarray. Science (1995) 270:467–70. 10.1126/science.270.5235.467 7569999

[13] Londoño MC Danger R Giral M Soulillou JP Sánchez-Fueyo A Brouard S. A need for biomarkers of operational tolerance in liver and kidney transplantation. Am J Transplant (2012) 12:1370–7. 10.1111/j.1600-6143.2012.04035.x 22486792

[14] Olson NE. The microarray data analysis process: from raw data to biological significance. NeuroRx (2006) 3:373–83. 10.1016/j.nurx.2006.05.005 16815220 PMC3593381

[15] Slonim DK Yanai I Getting started in gene expression microarray analysis. PLoS Comput Biol (2009) 5:e1000543 10.1371/journal.pcbi.1000543 19876380 PMC2762517

[16] Simon RM Dobbin K Experimental design of DNA microarray experiments. Biotechniques (2003) (Suppl):16–21. 12664680

[17] Naidu CN Suneetha Y Review article: current knowledge on microarray technology – an overview. Trop J Pharm Res (2012) 11:1–12. 10.4314/tjpr.v11i1.20

[18] Editorial. Making the most of microarrays. Nat Biotech (2006) 24:1039–1039. 10.1038/nbt0906-1039 16964193

[19] Kauffmann A Gentleman R Huber W. arrayQualityMetrics – a bioconductor package for quality assessment of microarray data. Bioinformatics (2009) 25:415–6. 10.1093/bioinformatics/btn647 19106121 PMC2639074

[20] Du P Kibbe WA Lin SM. lumi: a pipeline for processing Illumina microarray. Bioinformatics (2008) 24:1547–8. 10.1093/bioinformatics/btn224 18467348

[21] Luo W Gudipati M Jung K Chen M Marschke KB. Goober: a fully integrated and user-friendly microarray data management and analysis solution for core labs and bench biologists. J Integr Bioinform (2009) 6:108. 10.2390/biecoll-jib-2009-108 20134074

[22] Chatziioannou A Moulos P Kolisis FN. Gene ARMADA: an integrated multi-analysis platform for microarray data implemented in MATLAB. BMC Bioinformatics (2009) 10:354. 10.1186/1471-2105-10-354 19860866 PMC2771024

[23] Quackenbush J Computational analysis of microarray data. Nat Rev Genet (2001) 2:418–27. 10.1038/35076576 11389458

[24] Tárraga J Medina I Carbonell J Huerta-Cepas J Minguez P Alloza E GEPAS, a web-based tool for microarray data analysis and interpretation. Nucleic Acids Res (2008) 36:W308–14. 10.1093/nar/gkn303 18508806 PMC2447723

[25] Dinu I Potter JD Mueller T Liu Q Adewale AJ Jhangri GS Improving gene set analysis of microarray data by SAM-GS. BMC Bioinformatics (2007) 8:242. 10.1186/1471-2105-8-242 17612399 PMC1931607

[26] Bittner M Meltzer P Trent J Data analysis and integration: of steps and arrows. Nat Genet (1999) 22:213–5. 10.1038/10265 10391202

[27] Vingron M Bioinformatics needs to adopt statistical thinking. Bioinformatics (2001) 17:389–90. 10.1093/bioinformatics/17.5.389 11331232

[28] Simon R. Roadmap for developing and validating therapeutically relevant genomic classifiers. J Clin Oncol (2005) 23:7332–41. 10.1200/JCO.2005.02.8712 16145063

[29] Simon R Radmacher MD Dobbin K McShane LM Pitfalls in the use of DNA microarray data for diagnostic and prognostic classification. J Natl Cancer Inst (2003) 95:14–8. 10.1093/jnci/95.1.14 12509396

[30] Ioannidis JPA Khoury MJ. Improving validation practices in “omics” research. Science (2011) 334:1230–2. 10.1126/science.1211811 22144616 PMC5624327

[31] Penkett CJ Bähler J. Navigating public microarray databases. Comp Funct Genomics (2004) 5:471–9. 10.1002/cfg.427 18629145 PMC2447434

[32] Suter DM Molina N Gatfield D Schneider K Schibler U Naef F. Mammalian genes are transcribed with widely different bursting kinetics. Science (2011) 332:472–4. 10.1126/science.1198817 21415320

[33] Raghavan A Ogilvie RL Reilly C Abelson ML Raghavan S Vasdewani J Genome-wide analysis of mRNA decay in resting and activated primary human T lymphocytes. Nucleic Acids Res (2002) 30:5529–38. 10.1093/nar/gkf682 12490721 PMC140061

[34] Shen-Orr SS Tibshirani R Khatri P Bodian DL Staedtler F Perry NM Cell type-specific gene expression differences in complex tissues. Nat Meth (2010) 7:287–9. 10.1038/nmeth.1439 PMC3699332 20208531

[35] Hall N. Advanced sequencing technologies and their wider impact in microbiology. J Exp Biol (2007) 210:1518–25. 10.1242/jeb.001370 17449817

[36] Wetterstrand KA DNA Sequencing Costs: Data from The NHGRI Genome Sequencing Program (Gsp). Available from: www.genome.gov/sequencingcosts

[37] Wolf JBW. Principles of transcriptome analysis and gene expression quantification: an RNA-seq tutorial. Mol Ecol Resour (2013) 13:559–72. 10.1111/1755-0998.12109 23621713

[38] Scherer A. Clinical and ethical considerations of massively parallel sequencing in transplantation science. World J Transplant (2013) 3:62–7. 10.5500/wjt.v3.i4.62 24392310 PMC3879525

[39] Martinez-Llordella M Puig-Pey I Orlando G Ramoni M Tisone G Rimola A Multiparameter immune profiling of operational tolerance in liver transplantation. Am J Transplant (2007) 7:309–19. 10.1111/j.1600-6143.2006.01621.x 17241111

[40] Ashton-Chess J Giral M Brouard S Soulillou J-P. Spontaneous operational tolerance after immunosuppressive drug withdrawal in clinical renal allotransplantation. Transplantation (2007) 84:1215–9. 10.1097/01.tp.0000290683.54937.1b 18049104

[41] Pham MX Teuteberg JJ Kfoury AG Starling RC Deng MC Cappola TP Gene-expression profiling for rejection surveillance after cardiac transplantation. N Engl J Med (2010) 362:1890–900. 10.1056/NEJMoa0912965 20413602

[42] Ashton-Chess J Giral M Mengel M Renaudin K Foucher Y Gwinner W Tribbles-1 as a novel biomarker of chronic antibody-mediated rejection. J Am Soc Nephrol (2008) 19:1116–27. 10.1681/ASN.2007101056 18369086 PMC2396937

[43] Martínez-Llordella M Lozano J-J Puig-Pey I Orlando G Tisone G Lerut J Using transcriptional profiling to develop a diagnostic test of operational tolerance in liver transplant recipients. J Clin Invest (2008) 118:2845–57. 10.1172/JCI35342 18654667 PMC2483684

[44] Pons JA Yélamos J Ramírez P Oliver-Bonet M Sánchez A Rodríguez-Gago M Endothelial cell chimerism does not influence allograft tolerance in liver transplant patients after withdrawal of immunosuppression. Transplantation (2003) 75:1045–7. 10.1097/01.TP.0000058472.71775.7D 12698096

[45] Tisone G Orlando G Cardillo A Palmieri G Manzia T-M Baiocchi L Complete weaning off immunosuppression in HCV liver transplant recipients is feasible and favourably impacts on the progression of disease recurrence. J Hepatol (2006) 44:702–9. 10.1016/j.jhep.2005.11.047 16473433

[46] Kawasaki M Iwasaki M Koshiba T Fujino M Hara Y Kitazawa Y Gene expression profile analysis of the peripheral blood mononuclear cells from tolerant living-donor liver transplant recipients. Int Surg (2007) 92:276–86. 18399100

[47] Lozano JJ Pallier A Martinez-Llordella M Danger R López M Giral M Comparison of transcriptional and blood cell-phenotypic markers between operationally tolerant liver and kidney recipients. Am J Transplant (2011) 11:1916–26. 10.1111/j.1600-6143.2011.03638.x 21827613

[48] Bohne F Martínez-Llordella M Lozano J-J Miquel R Benítez C Londoño M-C Intra-graft expression of genes involved in iron homeostasis predicts the development of operational tolerance in human liver transplantation. J Clin Invest (2012) 122:368–82. 10.1172/JCI59411 22156196 PMC3248302

[49] Li L Wozniak LJ Rodder S Heish S Talisetti A Wang Q A common peripheral blood gene set for diagnosis of operational tolerance in pediatric and adult liver transplantation. Am J Transplant (2012) 12:1218–28. 10.1111/j.1600-6143.2011.03928.x 22300520

[50] Weiss G. Iron and immunity: a double-edged sword. Eur J Clin Invest (2002) 32(Suppl 1):70–8. 10.1046/j.1365-2362.2002.0320s1070.x 11886435

[51] Wang L Cherayil BJ. Ironing out the wrinkles in host defense: interactions between iron homeostasis and innate immunity. J Innate Immun (2009) 1:455–64. 10.1159/000210016 20375603 PMC3969595

[52] Girelli D Pasino M Goodnough JB Nemeth E Guido M Castagna A Reduced serum hepcidin levels in patients with chronic hepatitis C. J Hepatol (2009) 51:845–52. 10.1016/j.jhep.2009.06.027 19729219 PMC2761995

[53] Zhao X Li Y Ohe H Nafady-Hego H Uemoto S Bishop GA Intragraft Vδ1 γδ T cells with a unique T-cell receptor are closely associated with pediatric semiallogeneic liver transplant tolerance. Transplantation (2013) 95:192–202. 10.1097/TP.0b013e3182782f9f 23222896

[54] Li Y Koshiba T Yoshizawa A Yonekawa Y Masuda K Ito A Analyses of peripheral blood mononuclear cells in operational tolerance after pediatric living donor liver transplantation. Am J Transplant (2004) 4:2118–25. 10.1111/j.1600-6143.2004.00611.x 15575917

[55] Wei L Gong X Martinez OM Krams SM. Differential expression and functions of microRNAs in liver transplantation and potential use as non-invasive biomarkers. Transpl Immunol (2013) 29:123–9. 10.1016/j.trim.2013.08.005 24001411 PMC4031036

[56] Mas VR Dumur CI Scian MJ Gehrau RC Maluf DG MicroRNAs as biomarkers in solid organ transplantation. Am J Transplant (2012) 13:11–9. 10.1111/j.1600-6143.2012.04313.x 23136949 PMC3927320

[57] Danger R Pallier A Giral M Martínez-Llordella M Lozano J-J Degauque N Upregulation of miR-142-3p in peripheral blood mononuclear cells of operationally tolerant patients with a renal transplant. J Am Soc Nephrol (2012) 23:597–606. 10.1681/ASN.2011060543 22282590 PMC3312506

[58] Lorenzen JM Volkmann I Fiedler J Schmidt M Scheffner I Haller H Urinary miR-210 as a mediator of acute t-cell mediated rejection in renal allograft recipients. Am J Tansplant (2011) 11:2221–7. 10.1111/j.1600-6143.2011.03679.x 21812927

[59] Suthanthiran M Schwartz JE Ding R Abecassis M Dadhania D Samstein B Urinary-cell mRNA profile and acute cellular rejection in kidney allografts. N Engl J Med (2013) 369:20–31. 10.1056/NEJMoa1215555 23822777 PMC3786188

[60] Morita M Chen J Fujino M Kitazawa Y Sugioka A Zhong L Identification of microRNAs involved in acute rejection and spontaneous tolerance in murine hepatic allografts. Sci Rep (2014) 4:6649. 10.1038/srep06649 25323448 PMC5377586

[61] Wang Y Tian Y Ding Y Wang J Yan S Zhou L MiR-152 may silence translation of CaMK II and induce spontaneous immune tolerance in mouse liver transplantation. PLoS One (2014) 9:e105096. 10.1371/journal.pone.0105096 25133393 PMC4136864

[62] Dugast E Chesneau M Soulillou J-P Brouard S. Biomarkers and possible mechanisms of operational tolerance in kidney transplant patients. Immunol Rev (2014) 258:208–17. 10.1111/imr.12156 24517435

[63] Brouard S Mansfield E Braud C Li L Giral M Hsieh S-C Identification of a peripheral blood transcriptional biomarker panel associated with operational renal allograft tolerance. Proc Natl Acad Sci U S A (2007) 104:15448–53. 10.1073/pnas.0705834104 17873064 PMC2000539

[64] Newell KA Asare A Kirk AD Gisler TD Bourcier K Suthanthiran M Identification of a B cell signature associated with renal transplant tolerance in humans. J Clin Invest (2010) 120:1836–47. 10.1172/JCI39933 20501946 PMC2877933

[65] Pallier A Hillion S Danger R Giral M Racapé M Degauque N Patients with drug-free long-term graft function display increased numbers of peripheral B cells with a memory and inhibitory phenotype. Kidney Int (2010) 78:503–13. 10.1038/ki.2010.162 20531452

[66] Sagoo P Perucha E Sawitzki B Tomiuk S Stephens DA Miqueu P Development of a cross-platform biomarker signature to detect renal transplant tolerance in humans. J Clin Invest (2010) 120:1848–61. 10.1172/JCI39922 20501943 PMC2877932

[67] Braud C Baeten D Giral M Pallier A Ashton-Chess J Braudeau C Immunosuppressive drug-free operational immune tolerance in human kidney transplant recipients: part I. Blood gene expression statistical analysis. J Cell Biochem (2008) 103:1681–92. 10.1002/jcb.21574 17910029

[68] Braudeau C Ashton-Chess J Giral M Dugast E Louis S Pallier A Contrasted blood and intragraft toll-like receptor 4 mRNA profiles in operational tolerance versus chronic rejection in kidney transplant recipients. Transplantation (2008) 86:130–6. 10.1097/TP.0b013e31817b8dc5 18622290

[69] Danger R Thervet E Grisoni ML Puig PL Pallier A Tregouet D PARVG gene polymorphism and operational renal allograft tolerance. Transplant Proc (2012) 44:2845–8. 10.1016/j.transproceed.2012.09.034 23146538

[70] Bluestone JA Matthews JB The immune tolerance network – an NIH/JDF-supported initiative to bring tolerance research into the clinic: a major new resource for clinical immunologists. Clin Immunol (2000) 96:171–3. 10.1006/clim.2000.4892 10964534

[71] Louis SP Braudeau CC Giral M Dupont A Moizant FDR Robillard N Contrasting CD25hiCD4+T cells/FOXP3 patterns in chronic rejection and operational drug-free tolerance. Transplantation (2006) 81:398–407. 10.1097/01.tp.0000203166.44968.86 16477227

[72] Baron D Ramstein GER Chesneau MEL Echasseriau Y Pallier A Paul CE A common gene signature across multiple studies relate biomarkers and functional regulation in tolerance to renal allograft. Kidney Int (2015) 87:1–12. 10.1038/ki.2014.395 25629549 PMC4424816

[73] Khatri P Roedder S Kimura N De Vusser K Morgan AA Gong Y A common rejection module (CRM) for acute rejection across multiple organs identifies novel therapeutics for organ transplantation. J Exp Med (2013) 210:2205–21. 10.1084/jem.20122709 24127489 PMC3804941

